# Neonatal lupus erythematosus: an acquired autoimmune disease to be taken seriously

**DOI:** 10.1080/07853890.2025.2476049

**Published:** 2025-03-11

**Authors:** Wenqiang Sun, Changchang Fu, Xinyun Jin, Changda Lei, Xueping Zhu

**Affiliations:** a Department of Neonatology, Children’s Hospital of Soochow University, Suzhou, China; b Department of Pediatrics, Xinhua Hospital, Affiliated to Shanghai Jiao Tong University School of Medicine, Shanghai, China; c Department of Gastroenterology, First Affiliated Hospital of Soochow University, Suzhou, China

**Keywords:** Neonatal lupus erythematosus, autoimmune disease, autoantibody, congenital heart block, neurodevelopmental disorders

## Abstract

**Aim:**

This review aims to summarize the epidemiology, pathogenesis, clinical features, management, prognosis and regression of Neonatal lupus erythematosus (NLE) with a view to providing directions for standardized diagnosis, treatment and further research.

**Methods:**

We conducted a comprehensive literature review of NLE. NLE-related peer-reviewed papers were searched through PubMed/Medline were searched up to November 2024.

**Results:**

NLE is a rare acquired autoimmune disease (AD) linked to organ damage from maternal autoantibodies crossing the placenta to the foetus. However, not all mothers have ADs or associated antibodies. The disease involves autoantibody-induced inflammation, apoptosis, fibrosis, calcium channel dysregulation in cardiomyocytes, and increased interferon expression. NLE incidence shows no sex difference, but there is a differential distribution of clinical features across ethnic groups. The frequency of organ involvement in NLE patients is more common in the cutaneous and cardiac. NLE also affects the haematological and hepatobiliary systems, and some patients may experience neurological and endocrinological involvement. Steroids and immunoglobulins can aid in the recovery of some patients. Proper use of antimalarials during prenatal and gestational periods may prevent or improve the prognosis of patients with congenital heart block (CHB). Implantation of a pacemaker is effective in maintaining cardiac function in children with complete atrioventricular block. Symptoms associated with NLE may improve with antibody depletion, but some patients may experience sequelae such as irreversible CHB, neuropsychiatric disorders and developmental delays. Universal screening for autoantibodies to Sjögren syndrome A or B autoantigens should be offered to women of childbearing age experiencing desiccation syndrome. Antibody-positive individuals require appropriate reproductive counselling and advice, along with close foetal monitoring starting at 16 weeks of gestation and postnatal prognostic follow-up.

**Conclusion:**

Epidemiologic investigations and clinical studies on NLE are currently inadequate, and large-scale epidemiologic investigations, prospective clinical studies, and basic research are needed in the future to improve the understanding of the disease and the standardization of its clinical management.

## 1. Introduction

Immunoglobulin G (IgG) from mothers with rheumatic/autoimmune disease (AD) can cross the placenta, affecting foetal and post-natal [1,2]. In 1954, Bridge and Foley [3] first reported that maternal ‘lupus erythematosus factor’ could be transmitted to newborns. The understanding of neonatal lupus erythematosus (NLE) has improved with more reported cases. NLE is now recognized as an acquired AD, a clinical syndrome with multiple organ involvement induced by maternal IgG (mainly anti-SSA/Ro and anti-SSB/La antibodies) [4–6]. Most data on NLE come from observational studies and case reports, with few systematic summaries. The aim of this review is to summarize the epidemiology, pathogenesis, clinical features, management, prognosis and regression of NLE with a view to providing directions for standardized diagnosis, treatment and further research. For this purpose, we searched and screened the literature contained in databases such as PubMed/MEDLINE using the MeSH terms neonatal lupus or congenital heart block. Focus on reviewing representative reports and high-quality research papers. The search was performed from database construction to November 2024.

## 2. Epidemiology

NLE incidence is approximately 2% among offspring of mothers who are positive for anti-SSA and anti-SSB antibodies, increasing to 18–20% in subsequent pregnancies. Nearly 5% of patients with NLE may develop systemic lupus erythematosus (SLE) during adolescence or early adulthood [7–9]. The male-to-female ratio of patients with NLE and cutaneous involvement ranges from 2:1 to 3:1 [7–11]. However, subsequent studies indicate no significant difference between males and females in NLE incidence [7–9,11–15]. Some prospective studies found CHB incidence in the offspring of women positive for anti-SSA/Ro with uninvolved previous pregnancies at approximately 2%. However, in subsequent pregnancies, CHB incidence in offspring increases 10-fold [7–9,11,16–18].

Studies from Europe and America report congenital heart block (CHB) in nearly 70% of patients with NLE patients [7–9,11,19,20]. In Asia, less than 30% have cardiac involvement, but cutaneous involvement is twice that of patients from other regions. Studies show that 80% of patients with NLE in Asia have cutaneous involvement, with CHB incidence between 8.9% and 23%. However, one Japanese study found that approximately 50% of newborns with mothers who are anti-SSA/Ro antibody-positive developed CHB [7–9,11,21]. In lower middle income regions, missed pre-natal cardiac NLE diagnosis may increase estimates of spontaneous foetal losses of unknown aetiology. This leads to fewer documented CHB cases, further reducing its reported prevalence. It is easy to speculate whether there are maternal and infants genetics or other factors involved. Overall, however, universal screening and epidemiologic investigations of autoantibodies and prevalence of NLE in offspring in high-risk populations are currently inadequate, which may contribute to the wide variation in the prevalence of NLE reported.

The most common autoantibody detected in patients and mothers is anti-SSA/Ro, followed by anti-SSB/La antibodies. A small percentage may have anti-U1RNP antibodies. Among pregnant women positive for these autoantibodies, 0.1–1.5% were healthy, while 90% had Sjögren’s syndrome (SS), 20–30% had SLE, and 3% had rheumatoid arthritis (RA). Approximately 25% of mothers of patients with NLE show no autoimmune disease-related symptoms at delivery, with 50% developing symptoms within 3 years. Notably, 10–15% of mothers are negative for serum circulating autoantibodies [7–9,11,22,23]. In the review by Brito-Zeron et al. [24], the existence of ‘immunonegative’ CHB was questioned, as the vast majority of CHB cases are diagnosed in children ≥15 years of age or in mothers who have not been systematically assayed for CHB-associated autoantibodies. This challenge is very reasonable. It is well known that NLE is a syndrome caused by the transfer of circulating autoantibodies from the mother to the foetus *via* the placenta. In fact, asymptomatic women are usually not tested for autoantibodies; additionally, negative results on circulating antibody assays need to take into account differences in laboratory sample quality control and sensitivity.

## 3. Pathogenesis

The widely accepted idea is that NLE is mediated by maternal autoantibodies transferred through the placenta, which explains that the human foetus produces only trace amounts of IgA and IgM but not IgG, which is of maternal origin. In the third trimester, due to its small size, maternal IgG passes easily through the placenta, resulting in foetal IgG levels approximately 10% higher than maternal levels at full-term. At physiological pH, neonatal FcRs ensure maternal IgG release into the foetal circulation *via* endocytosis [1,2,7–9,11,25]. The IgG antibodies inducing NLE include mainly anti-SSA/Ro and anti-SSB/La. These enter the foetal circulation through the placenta, specifically targeting foetal tissue antigens, forming antigen-antibody complexes and activating inflammatory reactions, causing foetal tissue damage. Anti-SSA/Ro antibody targets four miRNAs 45, 52, 54 and 60 kDa) but only used both anti-SSA/Ro52 and anti-SSA/Ro60 clinically. Anti-SSB/La primarily recognizes a 47kD cytoplasmic protein. Ro52 is more likely to induce NLE-associated phenotypes in animal models and functions as a ubiquitin E3 ligase, promoting inflammatory responses and cell death. Additionally, it is a component of the TRIM21 receptor family, regulating T-cell activation and pro-inflammatory interleukin production [7–9,11,26–32].

Most mechanistic studies have focused on diseases with poor prognosis in patients with NLE and CHB. Anti-SSA/Ro antibodies are linked to an increased CHB risk in NLE [7–9,11,33–35]. High anti-SSB/La antibody levels may correlate with non-cardiac features [7,11,36]. Anti-SSA/Ro antibodies bind to foetal cardiomyocytes, inhibiting apoptotic cell clearance during foetal heart development, resulting in foetal or neonatal arrhythmias and structural cardiac. Additionally, antigen-antibody complex generation enhances Toll-like receptor signalling, triggers macrophage activation and fibrogenic factor secretion, and is associated with myocardial fibrosis development. At 18–26 weeks of gestation, type A or B autoantigens translocate to cardiomyocyte surfaces. Maternal IgG antibodies then enter foetal circulation, binding to these antigens and causing atrioventricular node injury. The cross-reactivity between cardiomyocyte L- and T-type calcium channels and autoantibodies disrupt cardiomyocyte calcium homeostasis and affect action potential conduction [7,11,24,32,37]. Related study shows that only foetuses exposed to high doses of maternal Anti-SSA/Ro antibodies are at risk for antibody-mediated heart disease [38,39]. Edgar Jaeggi et al. [38]used solid-phase enzyme-linked immunosorbent assay for maternal 60-kd Ro and 52-kd Ro proteins and found that only the offspring of mothers with Anti-SSA/Ro antibodies titres greater than 50 units/ml developed cardiac neonatal lupus. They then used chemiluminescent immunoassay and multiplex bead-based immunoassay to similarly confirm the association between high titres of Anti-SSA/Ro antibodies and the development of cardiac neonatal lupus [35]. Anti-U1RNP antibodies, associated with U1 spliceosome RNA, are linked to CHB development. When patients with CHB lack anti-SSA/Ro and anti-SSB/La antibodies and structural aetiology, testing for maternal anti-U1RNP antibody is advisable [29,32], and antibody titres may correlate with CHB development in patients with NLE. Mothers with high autoantibody titres face greater CHB risk in offspring than those with low titres [24,32]. The risk of NLE with CHB significantly increases when both anti-SSA/Ro and anti-SSB/La antibodies are positive [24]. CHB probability increases with the presence of anti-SSA/Ro, anti-SSB/La and anti-U1RNP antibodies [40].

Numerous studies suggest type I interferons (IFN) may be essential in CHB development. Neonates positive for both anti-SSA/Ro and anti-SSB/La antibodies exhibit increased expression of plasma IFN regulatory genes and IFNα [41,42]. Type I IFN activates tissue-resident, infiltrates macrophages and upregulates Ro52 expression, accelerating apoptosis and inducing a localized inflammatory response in the foetal heart [43,44]. Additionally, type I IFN directly contributes to arrhythmogenic effect [45–47].

Genetic factors may also contribute to NLE development. However, a multi-ancestral cohort study on anti-SSA/Ro antibody-positive found no association between SLE-associated genes and NLE outcome risk [48].

## 4. Clinical features and management of NLE

Clinical manifestations associated with NLE may appear at birth or within the first few weeks, usually before 4–6 weeks [10,13,15,49,50]. NLE often presents reversible symptoms of generalized multi-organ involvement and resolves as antibodies diminish, often within 6–12 months [51]. Cutaneous involvement is prevalent, followed by haematological, hepatobiliary and cardiac involvement; neurological and endocrinological involvement may occur in some patients [13,51,52].

### 4.1. Cutaneous

Cutaneous lesions are common in NLE, resembling those in children and adolescents with SLE. Relevant studies show varying rates of cutaneous involvement among ethnic groups, with Asians having the highest, up to 45.2%, overall and approaching 80% in some regions [15,22,52]. These usually appear at birth (20%) or within weeks (80%), with a cephalofacial distribution being the most common (up to 95%), followed by the trunk and extremities, respectively. Some patients may develop generalized cutaneous involvement. Ultraviolet exposure accelerates cutaneous lesions onset and progression [4,13,53,54]. Lesions primarily appear as macular rings or oval erythema with protruding edges, occasionally as papular or plaque-like lesions, some with central hyaline or fine-scaled lesions. Most lesions are usually 1 cm in diameter, with some fusing to form patchy erythema [50,55–57]. A small percentage of patients with NLE may present with oral mucosal lesions, mainly oral ulcers [58,59]. Cutaneous lesions typically resolve within six months in most patients and within one year in all patients [4,13,60]. Histological examination reveals granular IgG deposits at the dermal-epidermal junction and vesicular changes at the interface and skin adnexal structures [56].

Cutaneous lesions in NLE usually improve as antibodies deplete. Sunlight avoidance are the main treatment [61,62]. Topical steroids effectively treat cutaneous lesions in NLE but may not be necessary in all cases [58,62,63]. There have also been reports of reduced cutaneous rash in infants born to mothers taking hydroxychloroquine (HCQ) [64]. Some patients with NLE may experience long-term cutaneous damage, including capillary dilatation, hyperpigmentation and atrophic scars [63,65–67]. Cutaneous sequelae may be associated with photodamage, topical steroid use, immune complex deposition, etc. [65]. Patients with NLE who develop dilated capillaries can be treated with laser therapy. In conclusion, patients with NLE and cutaneous lesions require long-term follow-up with timely symptomatic interventions to minimize sequelae.

### 4.2. Haematological

NLE patients often experience isolated decreases in specific blood cell lineages, with anaemia being the most common, followed by thrombocytopenia and neutropenia. A small number of infants may present with pancytopenia resembling aplastic anaemia. However, precise estimates of prevalence are challenging since otherwise well newborns do not routinely undergo complete blood count testing. In contrast to the mechanism of lymphopenia in SLE, neutropenia in NLE is associated with the direct destruction of anti-SSA/Ro antibodies [68]. Zuppa AA et al. [51] found that clinical and laboratory monitoring of newborns born to anti-SSA/Ro antibody-positive mothers should be performed at 3 months of age, which is the time when infants have a high prevalence of haematologic and liver test abnormalities. Haematological symptoms usually resolve spontaneously, while symptomatic anaemia and thrombocytopenia may require blood transfusions [13,69,70]. Steroids/immunoglobulin therapy yields better results in some patients [7,71]. It’s worth noting that pancytopenia in NLE patients needs to be differentiated from familial hemophagocytic lymphohistiocytosis/aplastic anaemia, etc. [72–74]. Pancytopenia in NLE patients is usually transient, and some symptomatic children improve with transfusion therapy [51].

### 4.3. Cardiac

Cardiac involvement is common in patients with NLE, with rates varying by ethnicity, reaching approaching 70% in Europe and the Americas [19,20] and less than 30% in Asia [15,52]. This involvement includes chronic CHB, arrhythmias and myocarditis. Studies indicate patients with NLE have a higher incidence of structural heart defects than normal newborns [7,14,15]. However, a clear causal relationship and the exact mechanism remain unknown despite the absence of high-quality clinical studies on NLE and congenital cardiac structural defects.

The riskiest manifestation of cardiac involvement in NLE is CHB, occurring at a high rate, with a 15–20% risk of death from complete atrioventricular block (CAVB) [20,75]. It is believed that 60–90% of CHB cases are due to maternal antibodies entering the foetal circulation, unrelated to heart structural abnormalities [76]. CHB typically develops between the 18th and 26th weeks of gestation, less commonly after 26-30 weeks. Foetuses with severe CHB may exhibit bradycardia in utero, with a normal atrial and ventricular rate (40 –80/min [24,31,77,78]. Some newborns with severe CHB may develop myocardial dysfunction, possibly due to endocardial elastic fibre hyperplasia and myocardial fibrosis or associated with arrhythmia-induced cardiac dysfunction [24,31,77,78]. A related study confirmed that steroids and immunoglobulins completely improved biventricular dysfunction in NLE patients without arrhythmias [79]. A long-term follow-up study of 239 patients with NLE and cardiac involvement found that 2.4% developed cardiac insufficiency by age 1, 14.8% between ages 1 and 17, and 28.1% after age 17. Additionally, 43.8% of children with cardiac insufficiency by age 1 persisted until ages 1 to 17 [80]. Therefore, long-term follow-up is essential for patients with cardiac involvement.

CHB caused by NLE requires a wide range of perinatal and postnatal interventions. CHB induced by anti-SSA/Ro and anti-SSB/La antibodies is common and irreversible, causing a severe burden on patients and their families. In this population, anti-SSA/Ro antibody positivity ranged from 0.5% to 2.7%, and anti-SSB/La antibody positivity was 0.2% [81–83]. Currently there is wide variation in the timing of foetal cardiac monitoring in pregnant women with autoantibodies against SSA/Ro [84]. Mothers with autoimmune diseases, especially those positive for anti-SSA/Ro and anti-SSB/La antibody antibodies, should receive counselling about CHB risk during prenatal care. Per Canadian Rheumatology Association Recommendations, women with SLE should be tested for these antibodies before and during the first three months of pregnancy for timely monitoring and prophylactic treatment [85]. Regular ultrasonic cardiogram (UCG) monitoring is required to detect abnormalities, such as CHB, guiding delivery timing and treatment initiation. Foetal arrhythmias can be detected by UCG starting at 16–17 weeks of gestation, prompting regular screening of high-risk foetuses from the 16th week. Since few CHB cases are detected post-birth, monitoring during the first month after birth is also required for such patients [22,24,86,87]. Foetal heart rate and rhythm is effective in identifying atrioventricular block in the foetus and may reduce the need for serial echocardiograms, according to new research [34].

Studies have shown that the rational use of HCQ, an antimalarial drug, reduces cardiac involvement risk in children with NLE by 98.7%, possibly due to its cardioprotective effect in inhibiting toll-like receptor signalling [11]. HCQ significantly reduces the recurrence rate of CHB in women who previously delivered an affected infant, with a protective effect exceeding 50% [18,88]. The 2020 American College of Rheumatology Reproductive Health Management Guidelines for Rheumatoid and Musculoskeletal Diseases recommend HCQ treatment for all pregnant women positive for anti-SSA/Ro-and/or anti-SSB/La antibodies to reduce CHB risk [89]. Currently, dexamethasone and/or IVIG are commonly used to treat II and III foetal atrioventricular blocks [84,90]. However, Previous studies have shown conflicting results regarding the effectiveness of fluorinated steroid betamethasone and immunoglobulin in preventing cardiac involvement in patients with NLE [24,35,78,86,91]. The 2020 American College of Rheumatology Guidelines for the Management of Reproductive Health in Rheumatic and Musculoskeletal Diseases recommend 4 mg/day oral dexamethasone for pregnant women positive for anti-SSA/Ro and/or anti-SSB/La antibodies, with UCG suggesting CHB presence in the foetus [89]. Plasmapheresis may reverse and improve CHB but may be ineffective for CAVB [92].

Pacemaker implantation is an effective treatment option for patients with CAVB. Most require a pacemaker within one year of birth to maintain cardiac function [35,93–95]. After 9 years of follow-up, the survival of patients with CHB, although pacemaker implantation, can also induce abnormal electrical activation patterns [95,96].

### 4.4. Hepatobiliary

Hepatobiliary involvement in patients with NLE is mainly characterized by hepatomegaly, elevated transaminases, cholestasis, feeding intolerance and rarely, splenomegaly and peritoneal effusion [14,51,52,54,97–99]. This involvement is self-limiting, usually resolving within 6 months [14,54,70]. Manifestations associated with hepatobiliary involvement in NLE are often detected through laboratory tests. Relevant studies have shown that patients with NLE and hepatobiliary involvement often present with feeding intolerance and diarrhoea as the first manifestations, which may be attributed to lupus antibody-induced vascular inflammation throughout the hepatobiliary [14]. Therefore, offspring of mothers with AD or NLE-related antibodies who develop hepatobiliary involvement, such as feeding intolerance, after birth should be promptly examined and treated symptomatically. Some studies have suggested that steroids and immunoglobulins may effectively treat liver symptoms in patients with NLE [7]. However, this remains a subject for further research.

### 4.5. Neurological

Neurological involvement due to NLE is often overlooked but not uncommon. Central nervous system (CNS) involvement in patients with NLE can manifest as benign macrocephaly, seizures, cerebral haemorrhage and spinal cord lesions [13,49,100–103]. NLE-induced neurological involvement is usually asymptomatic and typically resolves without adverse sequelae once the antibodies subside. Intracranial haemorrhage may also be associated with antibody-induced systemic vasculitis. Lupus-associated antibodies can affect blood vessels throughout the body, often resulting in thrombosis, vasospasm, aneurysms and haemorrhage in the CNS [104,105]. Aseptic meningitis has also been reported in NLE, usually presenting with fever as the first symptom. CNS involvement may not be obvious, and brain magnetic resonance imaging findings may show irregular enhancement or no abnormalities. Hydrocephalus may occur in a small number of patients, and the diagnosis can be clarified through cerebrospinal fluid (CSF) analysis [13,49,69]. Steroids and immunoglobulins may be effective against aseptic meningitis [13].

Some studies have evaluated neurodevelopmental outcomes in patients with NLE. A prospective study from the United States [9] showed that 40% of children exposed to maternal autoantibodies developed neuropsychiatric disorders, significantly higher than the 27% in the control group. However, there were no significant differences between the two groups in the prevalence of depression, anxiety, developmental delays, learning, hearing and speech problems. In a related study, patients with CAVB associated with anti-SSA/Ro antibody positivity were followed up over an extended period, and neurodevelopmental assessment was performed using the Griffiths Developmental Assessment Scale and the Wechsler Intelligence Scale for Children. The intellectual level of the patients was normal, except for one patient with a learning disability [106]. However, it has also been shown that some patients with NLE and CNS involvement experience seizures and varying degrees of growth retardation, which usually improves with rehabilitation therapy [13,70]. Few relevant studies have addressed neurodevelopmental outcomes in NLE, and it is not yet completely clear whether there is a direct causal relationship between long-term neurodevelopmental delays or adverse neurological events and NLE in patients with NLE.

### 4.6. Endocrinological

A variety of endocrine glands, including the pancreas and thyroid, can be affected by ADs, and endocrine gland damage is also present in patients with NLE [13,107]. A retrospective study from China [13] found that 9 of 39 patients with NLE had different types of endocrinological impairments, among which abnormal pancreatic function was the most common. This was mainly characterized by hyperinsulinemia with hypoglycaemia, hypothyroidism, hypoadrenocorticism and lysineurine-protein intolerance. Nine patients with endocrinological involvement tested positive for anti-SSA/Ro antibodies. Endocrinological involvement due to NLE is often transient and does not require special management; however, close monitoring of the patient’s blood glucose level is required for hyperinsulinemia with hypoglycaemia. If necessary, exogenous glucose supplementation is required to avoid the organ damage caused by hypoglycaemia.

## 5. Prognosis

The effect of maternal ADs on offspring has been a focal point and hot topic in current research. Offspring of mothers with ADs have a significantly increased risk of developing ADs and neurodevelopmental disorders [71,108–110]. Therefore, long-term follow-up for patients with NLE is necessary. Less than 5% of patients with NLE may progress to SLE during adolescence or early adulthood [7,8]. Besides SLE, patients with NLE also have a significantly increased risk of developing other types of ADs in the future [71,111]. A long-term follow-up study of patients with NLE (*n* = 49) and their unaffected siblings (*n* = 45) found that six children previously diagnosed with NLE eventually received a definitive diagnosis of Ads. These included two cases of juvenile rheumatoid arthritis, one case of Hashimoto’s thyroiditis, one case of psoriasis and iritis, one case of diabetes and psoriasis, and one case of congenital hypothyroidism and nephrotic syndrome [111].

A small percentage of offspring from mothers with ADs have varying degrees of neuropsychiatric disorders and developmental delays [9,13,70,109,110]. Offspring of mothers with SLE are more likely to have neurodevelopmental disorders, possibly related to foetal exposure to autoantibodies and steroids [102,109,110,112]. However, steroids are degraded and inactivated as they pass through the placenta, with only 10% reaching the foetus. Further studies are needed to determine whether these factors are associated with an increased neurodevelopmental risk in offspring. A related study demonstrated that a subpopulation of anti-dsDNA antibodies, called anti-N-methyl-D-aspartate-receptor antibodies, induces apoptosis in foetal neurons, which is associated with the development of neuropsychiatric disorders [113]. Although many studies have shown that mothers with ADs have a significantly increased risk of ADs and neurodevelopmental disorders in their offspring, some studies have not found this phenomenon. However, these studies did not investigate the mechanism linking NLE to neurodevelopmental issues. Furthermore, selective reporting by families and care providers may introduce bias, affecting the perceived prevalence of this association. Future controlled, prospective, large-scale clinical follow-up studies are required.

## 6. Conclusion

Epidemiologic descriptions and surveys on the type, frequency and severity of clinical presentations in NLE patients are scarce. NLE presents with reversible symptoms of multiple organ involvement associated with maternal autoantibodies, often involving the cutaneous, cardiac, haematological and hepatobiliary in a small percentage of patients, as well as neurological and endocrinological involvement. There were no significant differences in sex prevalence; however, organ involvement varied according to race. The main mechanisms involved in the development of NLE are fetotransmitted antibody-induced inflammation, apoptosis, fibrosis, an imbalance in calcium channel homeostasis in cardiomyocytes and increased IFN expression. Genetics may also play a role in the development of NLE. Maternal and infant genetic factors may confound the NLE and outcomes association, but such studies are rare, and this will be a key area for future research.

Steroids and immunoglobulins have therapeutic effects in some patients; however, some controversy remains. The appropriate use of HCQ during the prenatal and gestational periods may prevent or improve the prognosis of patients with NLE and CHB. Patients with NLE have an increased incidence of ADs, and some may experience CHB, neuropsychiatric disorders, and developmental delays. Pregnant mothers who test positive for these antibodies should be provided with reproductive counselling, long-term management and follow-up. Most studies on NLE are retrospective descriptive studies or case reports/series; controlled, prospective, large-scale clinical prospective studies are needed in the future.

## Data Availability

Data sharing is not applicable to this article as no new data were created or analyzed in this study.
